# The Metastasectomy and Timing of Pulmonary Metastases on the Outcome of Osteosarcoma Patients

**DOI:** 10.4137/cmo.s531

**Published:** 2009-09-14

**Authors:** Yu-Min Huang, Chun-Han Hou, Sheng-Mou Hou, Rong-Sen Yang

**Affiliations:** 1 Departments of Orthopaedics, College of Medicine, National Taiwan University and Hospital, Taipei, Taiwan.; 2 Departments of Orthopaedics, Shuang Ho Hospital—Taipei Medical University, Taipei, Taiwan.

**Keywords:** osteosarcoma, metastasectomy, pulmonary metastases, survival rate

## Abstract

**Background:**

The author intended to clarify the therapeutic effect and prognostic factors of metastasectomy and timing of pulmonary metastases in osteosarcoma patents.

**Methods:**

Data was obtained retrospectively on all consecutive osteosarcoma patients from 1985 to 2005 in author’s institute. Fifty-two patients with pulmonary nodules were identified, including 24 patients undergoing pulmonary metastasectomy treatment. These patients were categorized into four groups: group 1, patients with lung metastases at the initial presentation; group 2, lung metastases identified during the period of pre-operative chemotherapy; group 3, lung metastases identified during period of the post-operative che motherapy; group 4, lung metastases identified after therapy for the primary osteosarcoma completed.

**Results:**

In our study, the 2-, 3-, and 5-year overall survival rates for 52 patients were 49%, 39% and 20%. The 2-year overall survival rates were 18% for group 1, 32% for group 3, and 70% for group 4 (p < 0.001). The 5-year overall survival rate was 34% for group 4. Patients who underwent metastesectomy showed a better survival outcome as compared with the patients not undergoing metastasectomy (p = 0.003). The 2-year and 5-year overall survival rates of only one lung metastatic nodule were 62% and 50%, and for initially multiple lung metastatic nodules, 45% and 5%, respectively. In addition, the patients presented with lung metastases had a worse prognosis as compared with those without initial lung metastases (p = 0.0001).

**Conclusions:**

The patients having single metastatic nodule showed a better prognosis than those with multiple lung nodules. Furthermore, those patients who underwent metastasectomy survived longer than those not undergoing metastasectomy. Patients who had late metastases after complete chemotherapy had a better prognosis; whereas those who had metastases identified at the initial presentation predicted a poor prognosis.

## Introduction

Osteosarcoma is the most common malignant bone sarcoma in the children and adolescents. The long-term survival of patients with osteosarcoma has dramatically improved over the last two decades because of the combination of chemotherapy, local treatment and aggressive metastasectomies.^1^–^3^ The osteosarcoma patients without lung metastases had a 5-year survival rate around 65%–75%.^4^–^8^ However, pulmonary metastases occurs in approximately half of the osteosarcoma patients and 30% to 50% of the osteosarcoma patients still die of pulmonary metastasis.^2^,^3^,^9^–^11^ The 5-year event-free survival rate of the patients with lung metastases was dramatically decreased, although it was improved from 0%–17% to 30%–50% over the last 2 decades.^9^,^12^–^24^ Previous reports showed that approximately 15% to 20% of patients with osteosarcoma present with lung metastasis at initial diagnosis^14^,^25^–^27^ and more than 75% of synchronous metastatic lesions are found in the lungs.^14^,^16^,^17^,^28^ Therefore, treatment of the osteosarcoma patients with pulmonary metastases remains a big challenge at present.

The pulmonary metastectomy, i.e. resection of the pulmonary metastatic nodules may improve prognosis of these patients.^9^,^17^,^22^,^24^,^29^,^30^ The purpose of our study was to investigate the effect of timing of pulmonary metastases and the effect of metastasectomy in the survival of the osteosarcoma patients. We report a retrospective study of 52 osteosarcoma patients with pulmonary metastases at our institute in the past 20 years.

## Materials and Methods

From January 1985 to July 2005, 52 patients having osteosarcoma and lung metastases were treated in the authors’ institution. The patient’s average age was 17.5 years (range, 8 to 56 years) with 35 males and 17 females. Twenty-seven patients had primary tumor located in the femur, 16 in the tibia, 6 in the humerus, and 2 in the femoral neck and 1 in the scapula. The mean follow-up duration was 28.6 months (range 4 to 129 months). Of all the patients, 24 patients underwent metastasectomy of lung metastatic nodules. Among them, four patients had lung metastases proved by chest plain radiography or computed topography at the initial clinical presentation. We also identified the number of pulmonary nodules and classified them as single (7 patiens) or multiple lesions(17 patients). The other 28 patients did not receive pulmonary metastasectomy, and four of them were diagnosed with lung metastases at the initial clinical presentation. Twelve patients had single and 16 patients had multiple lung nodules.

**Figure 1.**
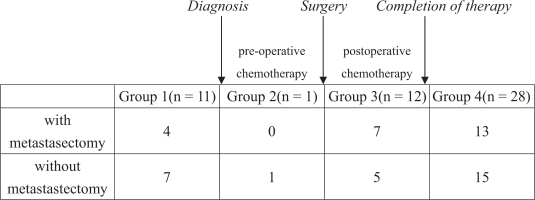
A demographic data of the patients in our study. These patients were divided into four groups according to the time of presenting lung metastases. Group 1, patients with initial lung metastases at presentation, group 2, lung metastases identified during the pre-operative chemotherapy period, group 3, lung metastases identified during the post-operative chemotherapy period, group 4, lung metastases identified after complete chemotherapy for the primary osteosarcoma.

In order to investigate the effect of timing of pulmonary metastases on the survival, we divided our 52 patients into four groups according to the time of lung metastases identification: group 1, 11 patients with lung metastases at the initial presentation; group 2, 1 patient with lung metastasis noted during the pre-operative chemotherapy period; group 3, 12 patients with lung metastases identified during the post-operative chemotherapy period; group 4, 28 patients with their lung metastases identified after complete chemotherapy for the primary osteosarcoma (Fig. 1A). In addition to the timing of lung metastases and metastasectomy, we retrospectively investigated several prognostic factors including primary tumor site, and the number of pulmonary metastatic lesions at initial presentation. Chest surgeons were consulted for further evaluation and management. The criteria for metastasectomy include (1) primary tumor under control, (2) no other metastatic lesions except lung, (3) adequate residual lung volume. After discussion with the patient and the family, we suggested metastasectomy if the patients are suitable to undergo surgery. Unfortunately, most patients didn’t undergo metastasectomy due to poor response to chemotherapy (rapid progression of disease) or complications of chemotherapy (neutropenic fever or sepsis).

The limb salvage operation was the preferred procedure to treat osteosarcoma patients. Most patients received preoperative chemotherapy, operation, and postoperative chemotherapy as scheduled. The major regimen of chemotherapy included cycles of cisplatin plus epirubicin or adramycin, etoposide plus cyclophospamid or ifosphamide. High-dose methotrexate (8 g/m^2^ for patients older than 12 years, 12 g/m^2^ for youger patients) was given with oral leucovorin rescue. The dose or regimen of chemotherapy would adjust according to the response of chemotherapy and the patients’ compliance. Lung metastatic lesion would be removed after complete therapy of primary osteosarcoma lesion. All osteosarcoma patients receiving metastasectomy had further chemotherapy.

Survival curve was analyzed by the method of Kaplan and Meier, and the significance of difference among groups were analyzed by log-rank test. A p-value of less than 0.05 was considered significant.

## Results

The 2-, 3-, and 5-year overall survival rates for these 52 patients were 49%, 39% and 20% (Fig. 2A). Figure 2B showed the patients with lung metastases at initial presentation (Enneking stage IIIB) had a worse prognosis than those without initial lung metastases (Enneking stage IIB) (p = 0.017). The patients without initial lung metastasis showed a better survival rate and it could be recognized as a prognostic factor. The overall 2-year survival rates were 18% for group 1, 0.1% for group 2, 32% for group 3, and 70% for group 4. The 5-year survival rate was 34% for group 4 patients (Fig. 2C). Only group 4 patients (lung metastases after complete therapy) can survive for more than 5 years, and they had a significantly better prognosis than the patients of other groups (p < 0.001). Figure 2D showed the relationship of the number of lung metastatic nodules on the survival rate of the patients with osteosarcoma. The patients with single metastatic nodule showed a statistically significant better prognosis than those patients with multiple lung nodules at presentation (p = 0.017). The 2-year and 5-year overall survival rates of single lung metastatic nodule were 62% and 50%, respectively. The 2-year and 5-year overall survival rate of initial multiple lung metastatic nodules were 45% and 5%. The number of lung metastatic nodules at initial presentation is a prognostic factor for osteosarcoma patients.

The most common sites of osteosarcoma were distal femur, proximal tibia and proximal humerus. Patients with primary osteosarcoma at distal femur and proximal tibia showed a better prognosis than those with tumor located in other sites (Fig. 2E). It was statistically significant if the patients can survive more than 5 years (p = 0.026).

Patients who underwent metastasectomy showed a significantly better survival outcome (Fig. 3A) (p < 0.05). We analyzed the survival outcomes the patients either undergoing metastasectomy or not separately. Among all four groups, the group 4 patients had the best survival prognosis, no matter undergoing metastasectomy (p = 0.0081) or not undergoing metastasectomy (p = 0.0006) (Fig. 3B). Furthermore, if we analyzed effects of metastasectomy in the patients at different time of presentation of lung metastases in these four groups, we still can find a trend that patients who underwent metastasectomy had a better prognosis (Fig. 3C).

## Discussion

The recent advance in the chemotherapy, aggressive surgical treatment and image studies improved the survival outcomes and functions in the patients with osteosarcoma. Bacci et al reported their 5-year and 10-year survival of these patients as 66% and 57%, respectively.^31^ However, half of these patients still develop lung metastases and the survival outcome was much worse as compared with those without lung metastases. From January 1985 to July 2005, there were two hundred and ten osteosarcoma diagnosed in our institute. Among them, only four pelvic (2%) osteosarcoma were found. In our study, these four patients didn’t develop pulmonary metastases. Similar results had been presented by Sabb et al Cancer Apr 1, 2005, Volume 103.^7^,^32^ Among 442 osteosarcoma patients, 19 patients (4%) had primary tumor in the pelvic bones. Only 4 patients developed pulmonary metastases in their groups.

The 5-year overall survival rate in the osteosarcoma patients with lung metastases was 20% in our series. However, these survival outcomes may still give hope and encouragement to try a more chemotherapy and aggressive treatment for those patients with pulmonary nodules. However, the patients with pulmonary metastases after complete therapy (group 4) can have a 2-year survival rate 82% and 5-year survival rate, 55%. Even the group 4 patients without metastasectomy still had a 2-year and 5-year survival rate up to 52% and 18%. It implies that a more aggressive treatment policy should be considerated. The survival of nonmetastatic osteosarcoma patients showed a dramatically better prognosis.^8^ However, we can find better prognostic factors in pulmonary metastasic patients, such as sites, the number of pulmonary nodule, staging, metastasectomy, and the timing of pulmonary metastases presentation. Hawkins et al also identified that the factors improving survival rates were unilateral pulmonary recurrence, solitary pulmonary nodules at recurrence, more than 24 months between the initial diagnosis and first disease recurrence, and achievement of a second complete response.^33^

Many reports advocated that a complete metastasectomy is a better predictor factor of survival in osteosarcoma patients with pulmonary metastases. 9,10,14,19 Even repeated thoracotomy for recurrent pulmonary metastases has been proved beneficial to patients.^17^,^34^ In our series, most patients underwent multiple surgeries for pulmonary metastatic nodules. We also obey the principles to treat the metastatic lung nodules. We suggested that metastasectomy may present as a good prognostic factor in osteosarcoma patients. If we perform as more aggressive surgery for lung metastatic nodules as possible, we can increase the survival rate even the patients with lung metastases. Briccoli et al^34^ also proposed that we should perform re-operation for recurrent pulmonary metastases due to similar disease-free interval after five years. In their study, the 3- and 5-year event-free survival rate was 45% and 38%, respectively; whereas from the second metastasecotmy, it was 33% and 32%, respectively. A study done by Snyder et al^35^ also supported that aggressive thoracotomy from bone and soft-tissue sarcoma should be considered when there is control of local disease, no evidence of extrapulmonary metastasis, and adequate postresection pulmonary reserve. They even performed thoracotomy on patients with bilateral, extensive, or recurrent pulmonary metastasis.

The effect of timing of pulmonary identification remains equivocal. Some studies showed that the patients having initial presentation of lung metastases had worse prognosis,^13^,^14^,^36^–^39^ and other studies do not show the same relationship. For example, Yonemoto et al^22^ claimed that the 5-year survival rate of osteosarcoma with pulmonary metastasis at initial presentation was 64.8%, which is not different from patients without pulmonary metastasis at initial presentation (62.1%). He also held that the studies with poor prognosis is due to inconsistent treatment. More aggressive chemotherapy and surgery should improve the survival.

In our study, the patients with lung metastases after complete therapy (Group 4) showed the best prognosis. Tsuchiya et al^40^ classified his patients with the same criteria we used in this study. He reported overall a 5-year survival rates of 18% for group 1, 0% for group 2, 6% for group 3, and 31% for group 4. Their group 4 patients also demonstrated a better prognosis than other groups.

We also find that the patients with lung metastases at initial presentation did not show the worst prognosis, as compared with the patients in group 2 and group 3. It may be due to the activation of angiogenic activity after resection of osteosarcoma tumor.^41^ It may remind us that a new treatment or more aggressive chemotherapy protocols, e.g. antiangiogenesis therapy, should be applied to those patients with pulmonary metastases when chemotherapy is ongoing. However, we still need to keep in mind the potential sources of bias due to the heterogeneity of the subgroups and the small size of the sample.

In conclusion, this study ensures that the good prognostic factors among the osteosarcoma patients with lung metastases were: 1. primary tumor sites at distal femur and proximal tibia, 2. staging IIB, 3. metastasectomy, 4. pulmonary metastases identified after complete treatment. According to our data, the close follow-up of the patients with osteosarcoma during the chemotherapy period is mandatory due to the possibility of the development of new lesions in lungs. The effect of metastasectomy has also been shown in this study. we recommended that osteosarcoma patients with pulmonary metastatic nodules, even for those with worse prognostic factors, should receive further chemotherapy as well as more aggressive surgery.

## Figures and Tables

**Figure 2A.**
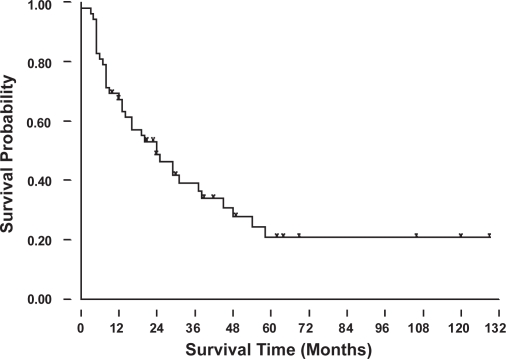
Overall survival rate for the osteosarcoma patients with pulmonary metastases.

**Figure 2B.**
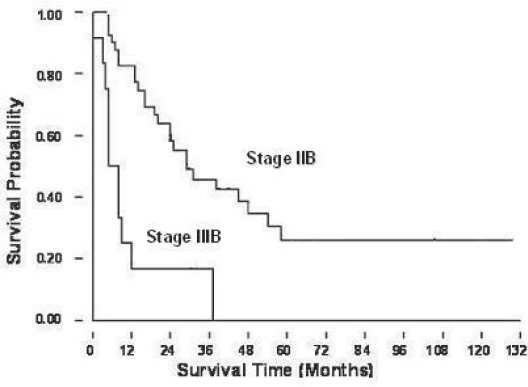
Patients with Enneking stage IIIB (lung metastasis) at initial presentation had a worse survival prognosis than those with IIB osteosarcoma.

**Figure 2C.**
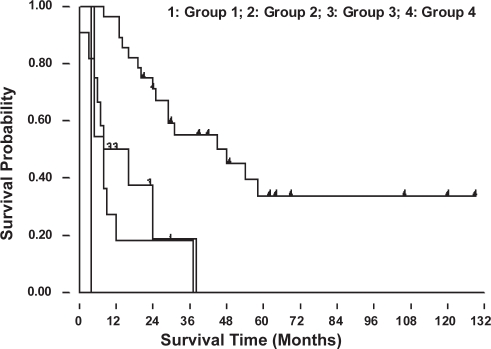
Overall survival probability for osteosarcoma patients according to the timing of pulmonary metastses. Group 4 patients had the best prognosis.

**Figure 2D.**
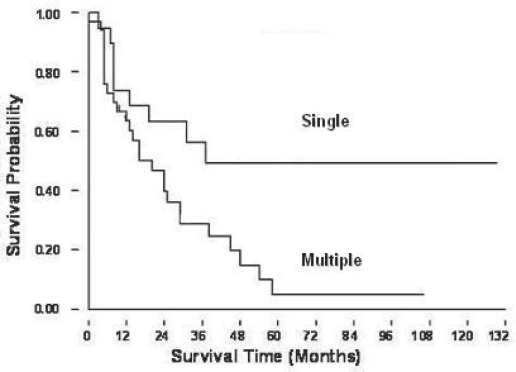
Patients with single metastatic lung nodule had a better survival prognosis as compared with those with multiple lung nodules

**Figure 2E.**
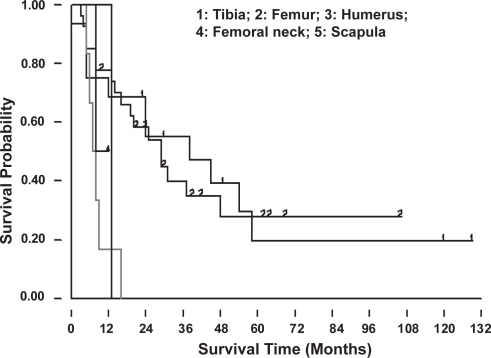
Cumulative survival curves for the primary tumor sites showed better results for those with the tumor located at the distal femur and proximal tibia. (Group 1: proximal tibia, group 2: distal femur, group 3: upper extremity, group 4: femoral neck; group 5: scapula).

**Figure 3A.**
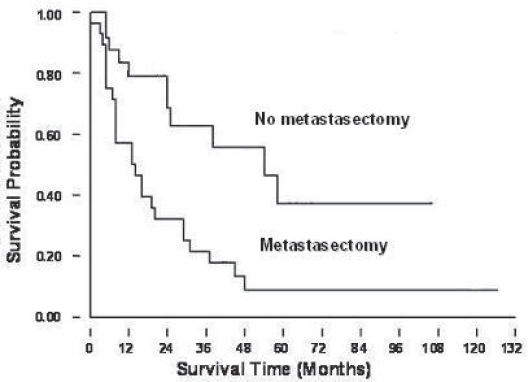
Kaplan-Meier survival curves showed better prognosis in the patients with metastasectomy., when comparing with patients who did not receive metastasectomy.

**Figure 3B.**
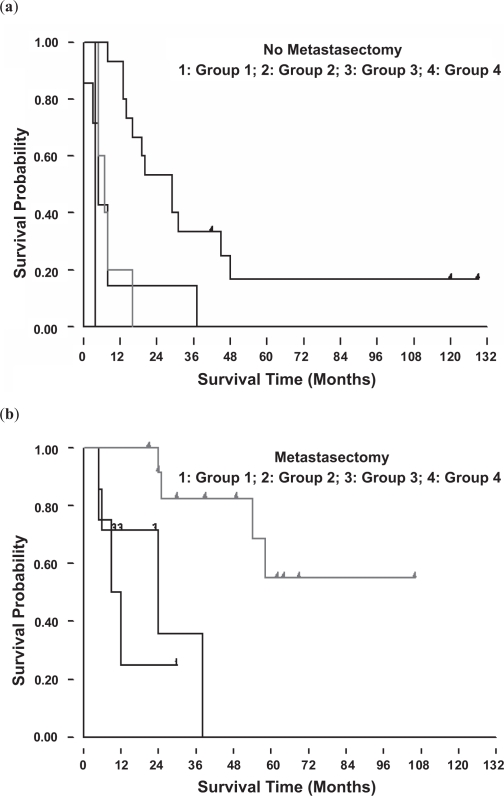
Survival for each group according to the timing of pulmonary metastases without (**a**) and with (**b**) metastasectomy. Group 4 patients had a better prognosis than the patients in the other groups.

**Figure 3C.**
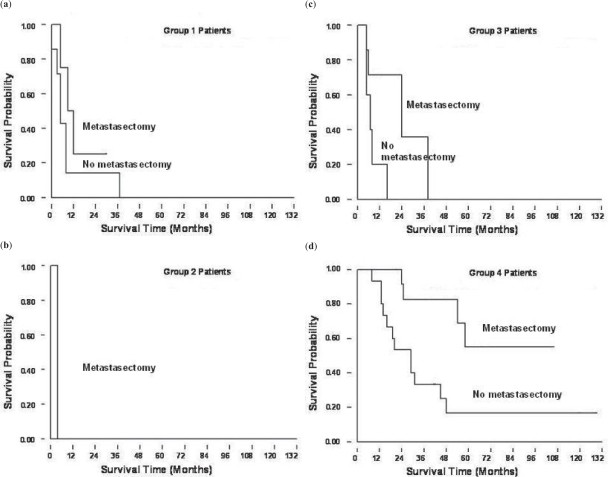
(**a**)~(**d**) represents group 1 to 4 patients respectively. In every group, patients who received metastasectomy had a better survival prognosis.
